# Avian coronavirus IBV-induced activation of the NLRP3–Caspase-1–IL-1β axis in renal collecting ducts contributes to nephropathogenesis

**DOI:** 10.1128/jvi.01466-25

**Published:** 2025-12-03

**Authors:** Min Huang, Chengyin Liukang, Rong Liang, Yingfei Li, Ruihua Yang, Mingwei Zhang, Ye Zhao, Jing Zhao, Guozhong Zhang

**Affiliations:** 1 State Key Laboratory of Veterinary Public Health and Safety, College of Veterinary Medicine, China Agricultural University 630101 , Beijing, People’s Republic of China; 2 Key Laboratory of Animal Epidemiology of the Ministry of Agriculture, College of Veterinary Medicine, China Agricultural University 630101 , Beijing, People’s Republic of China; University of Kentucky College of Medicine, Lexington, Kentucky, USA

**Keywords:** infectious bronchitis virus, NLRP3 inflammasome, renal inflammation, collecting duct epithelial cells, inflammation-mediated pathology

## Abstract

Avian coronavirus infectious bronchitis virus (IBV) induces severe renal inflammation and urate deposition in chickens, but the underlying mechanisms remain incompletely understood. Here, we used both *in vivo* chicken infection models and *in vitro* primary renal epithelial cell cultures and found that IBV activates the NLRP3 (NOD-like receptor family, pyrin domain containing 3) inflammasome, leading to the maturation and secretion of IL-1β (interleukin-1 beta). Pharmacological inhibition of NLRP3 in an *in vivo* chicken model alleviates renal pathology without affecting viral replication, implicating host inflammatory responses in disease progression. These findings indicate that host inflammasome activation, rather than direct viral replication or cytotoxicity, plays a central role in IBV-associated renal pathology. Notably, we observed that NLRP3 is predominantly expressed in the renal collecting duct, primarily within AQP2 (Aquaporin-2)-positive epithelial cells—an anatomic localization of inflammasome activity not previously reported. Single-cell transcriptomic profiling further revealed that IBV infection reprograms collecting duct cells from an electrolyte-regulating phenotype into a pro-inflammatory state, accompanied by disrupted urate metabolism and impaired ion transport. These findings identify collecting duct-targeted NLRP3 activation as a central mechanism of IBV-induced nephropathy and provide a theoretical foundation for developing targeted anti-inflammatory therapies.

**IMPORTANCE:**

*In vivo* studies in chickens demonstrate that the activation of the NLRP3 inflammasome is a key driver of renal injury during infectious bronchitis virus (IBV) infection. Pharmacological inhibition of NLRP3 significantly alleviates renal inflammation and tissue damage without affecting viral replication, highlighting the central role of host inflammatory responses in disease progression. Importantly, we report for the first time that NLRP3 activation is predominantly localized to AQP2-positive collecting ducts, a nephron segment essential for uric acid excretion and electrolyte balance. IBV infection reprograms these epithelial cells into a pro-inflammatory, metabolically dysregulated state, promoting urate crystal formation and amplifying tissue injury. These findings reveal a spatially confined epithelial-immune axis of coronavirus-induced renal pathology and suggest new avenues for targeted intervention.

## INTRODUCTION

Infectious bronchitis virus (IBV) is an enveloped, positive-sense single-stranded RNA virus and one of the earliest identified coronaviruses in the genus Gammacoronavirus (1, 2). In addition to its canonical respiratory manifestations, the currently predominant GI-19 IBV strains can cause acute nephritis with tubular lesions and urate deposition, particularly severe in young chickens (3). Although the clinical manifestations and histopathological characteristics of IBV infection have been well characterized (4), the underlying molecular mechanisms driving renal inflammation and tissue damage remain incompletely understood.

The innate immune system represents the host’s primary defense against viral infection, in which members of the NOD-like receptor (NLR) family, a group of nucleotide-binding domain and leucine-rich repeat-containing receptors, play a pivotal role in sensing intracellular danger signals, including invading viruses (5). Among them, the NLRP3 (NOD-like receptor family, pyrin domain containing 3) inflammasome is particularly critical. The NLRP3 inflammasome is a cytosolic multiprotein complex classically activated through the canonical “two-signal model” (6). The initial “priming” signal upregulates the transcription of NLRP3 and key pro-inflammatory mediators, including pro-IL-1β, via pattern recognition receptor pathways, particularly Toll-like receptors (TLRs) (7). The subsequent “activation” signal, elicited by cellular stressors such as ionic imbalance, mitochondrial dysfunction, or lysosomal rupture, facilitates NLRP3 oligomerization and the formation of the active inflammasome complex (8). Upon maturation, the inflammasome activates Caspase-1, which mediates the cleavage and secretion of the pro-inflammatory cytokines IL-1β and IL-18 (interleukin-18), ultimately driving robust inflammatory responses (7). Although inflammasome activation is central to antiviral immunity, dysregulated or sustained activation can trigger a cytokine storm, leading to severe tissue damage and immune-mediated pathology (9, 10). Notably, excessive inflammasome activation has been implicated in the pathogenesis of severe lung injury and increased mortality associated with severe acute respiratory syndrome coronavirus 2 (SARS-CoV-2) infection (11). Moreover, viral infections such as those caused by severe acute respiratory syndrome coronavirus (SARS-CoV), Middle East respiratory syndrome coronavirus (MERS-CoV), and influenza A virus (IAV) are associated with cytokine overproduction, including TNF-α, IL-1β, and type I/II interferons, which disrupt immune homeostasis and contribute to host tissue pathology (12–14). Clinical and experimental studies have shown that IBV infection is associated with marked upregulation of IL-1β in renal tissue, suggesting a potential role for the NLRP3 inflammasome signaling axis in IBV-induced renal injury (15, 16). However, the mechanisms by which IBV induces kidney inflammation and the contribution of the NLRP3 inflammasome to this process have yet to be characterized.

In this study, we investigated the role of the NLRP3 inflammasome in IBV-induced kidney injury using an *in vivo* chicken infection model and *in vitro* primary renal epithelial cell culture. Our results demonstrate that IBV infection induces NLRP3 activation in renal tissues, particularly within collecting duct epithelial cells. This localized inflammasome activity is accompanied by pro-inflammatory transcriptional reprogramming, altered urate metabolism, and impaired ion transport, contributing to urate crystal formation and exacerbation of tissue damage. Pharmacological inhibition of NLRP3 significantly attenuates renal inflammation and histological injury without affecting viral replication. These findings provide new insight into the cellular and molecular mechanisms underlying IBV-induced nephritis and highlight the collecting duct as a central site of immune-metabolic dysfunction during coronavirus infection.

## RESULTS

### IBV infection promotes cytokine-mediated renal inflammation in chickens

**Fig 1:**
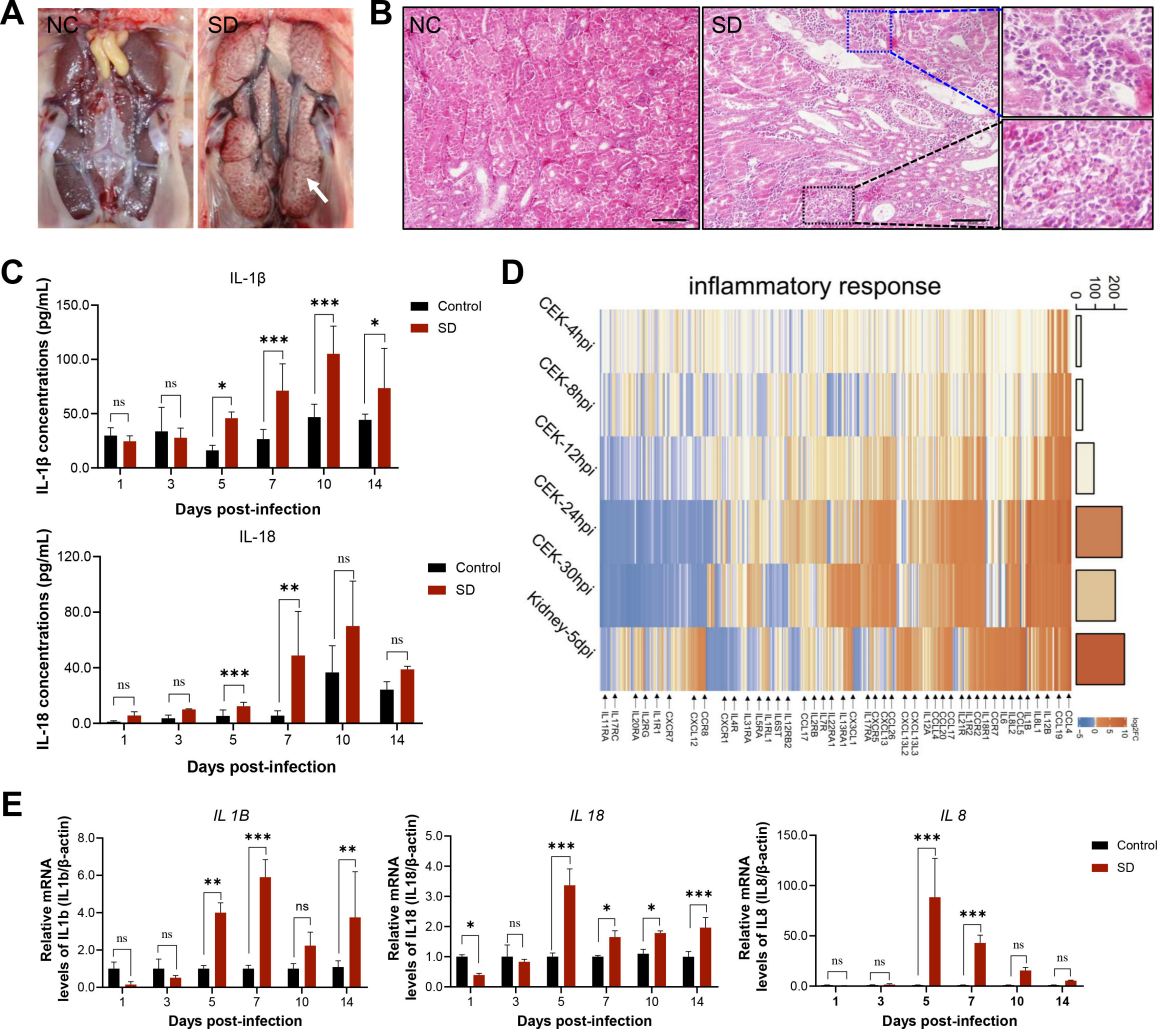
Renal inflammatory responses induced by IBV infection. (**A**) Gross pathology of kidneys at 5 dpi with the IBV SD strain. Infected kidneys show enlargement, pallor, and urate deposition (white arrowheads), whereas control kidneys appear grossly normal. (**B**) Histopathological analysis of renal tissues at 5 dpi (H&E staining). Control samples display intact tubular architecture, whereas IBV-infected kidneys exhibit vacuolar degeneration, tubular epithelial cell necrosis and desquamation (black boxes), and inflammatory infiltration in the renal interstitium (blue boxes). Scale bar = 50 µm. (**C**) Quantification of serum IL-1β and IL-18 protein levels by ELISA at multiple time points post-infection. (**D**) Heatmap displays the log_2_(fold change) of differentially expressed genes (DEGs) in IBV-infected CEK cells (at 4, 8, 12, 24, and 30 hpi) and kidney tissue (at 5 dpi) compared to mock controls. Upregulated and downregulated genes are depicted in shades of orange and blue, respectively. The adjacent bar plot quantifies the cumulative fold change of all DEGs for each sample. (**E**) qRT-PCR analysis of temporal expression patterns of selected pro-inflammatory cytokines in kidney tissues at 1, 3, 5, 7, 10, and 14 dpi. Expression levels were normalized to β-actin and expressed as relative fold changes. Data are expressed as mean ± SD, *n* = 3. Statistical significance: ns, not significant; *, *P* < 0.05; **, *P* < 0.01; ***, *P* < 0.001.

To investigate the molecular basis of IBV-induced renal pathology, we established an animal model by infecting 1-day-old specific-pathogen-free (SPF) chickens with IBV. Gross examination of kidneys from IBV-infected chickens revealed marked renal enlargement and urate deposition (Fig. 1A). Histopathological assessment further revealed severe renal lesions, characterized by widespread vacuolar degeneration, necrosis, and desquamation of renal tubular epithelial cells, as well as a prominent infiltration of inflammatory cells within the renal interstitium (Fig. 1B). These findings collectively indicated that IBV infection results in profound structural and cellular damage to renal tissues.

In order to characterize the inflammatory responses associated with IBV-induced renal injury, we quantified the expression of pro-inflammatory cytokines in serum. Enzyme-linked immunosorbent assay (ELISA) demonstrated a significant and sustained increase in circulating IL-1β and IL-18 levels during 5–14 days post-infection (dpi), consistent with a systemic inflammatory response (Fig. 1C). To gain a comprehensive view of host transcriptional changes, we performed high-throughput RNA sequencing (RNA-seq) on kidney tissues and primary chicken embryonic kidney (CEK) cells following IBV infection. Differential gene expression analysis revealed robust upregulation of a broad range of inflammation-associated genes (Fig. 1D), including pro-inflammatory interleukins (e.g., *IL1B*, *IL6*, and *IL12A*), chemokines (*CCL5 *and *CXCL13*), and cognate receptors (*IL1R1* and *CXCR5*), suggesting the activation of multiple inflammatory signaling pathways. These transcriptomic changes were further corroborated by quantitative reverse transcription PCR (qRT-PCR), which confirmed significant induction of *IL1B*, *IL18*, and *IL8* expression in renal tissues (Fig. 1E). These findings indicate that IBV infection induces renal inflammatory responses both *in vivo* and *in vitro*, which are associated with the upregulation of pro-inflammatory cytokines and chemokines.

### IBV infection induces NLRP3 inflammasome activation in chicken kidneys

**Fig 2:**
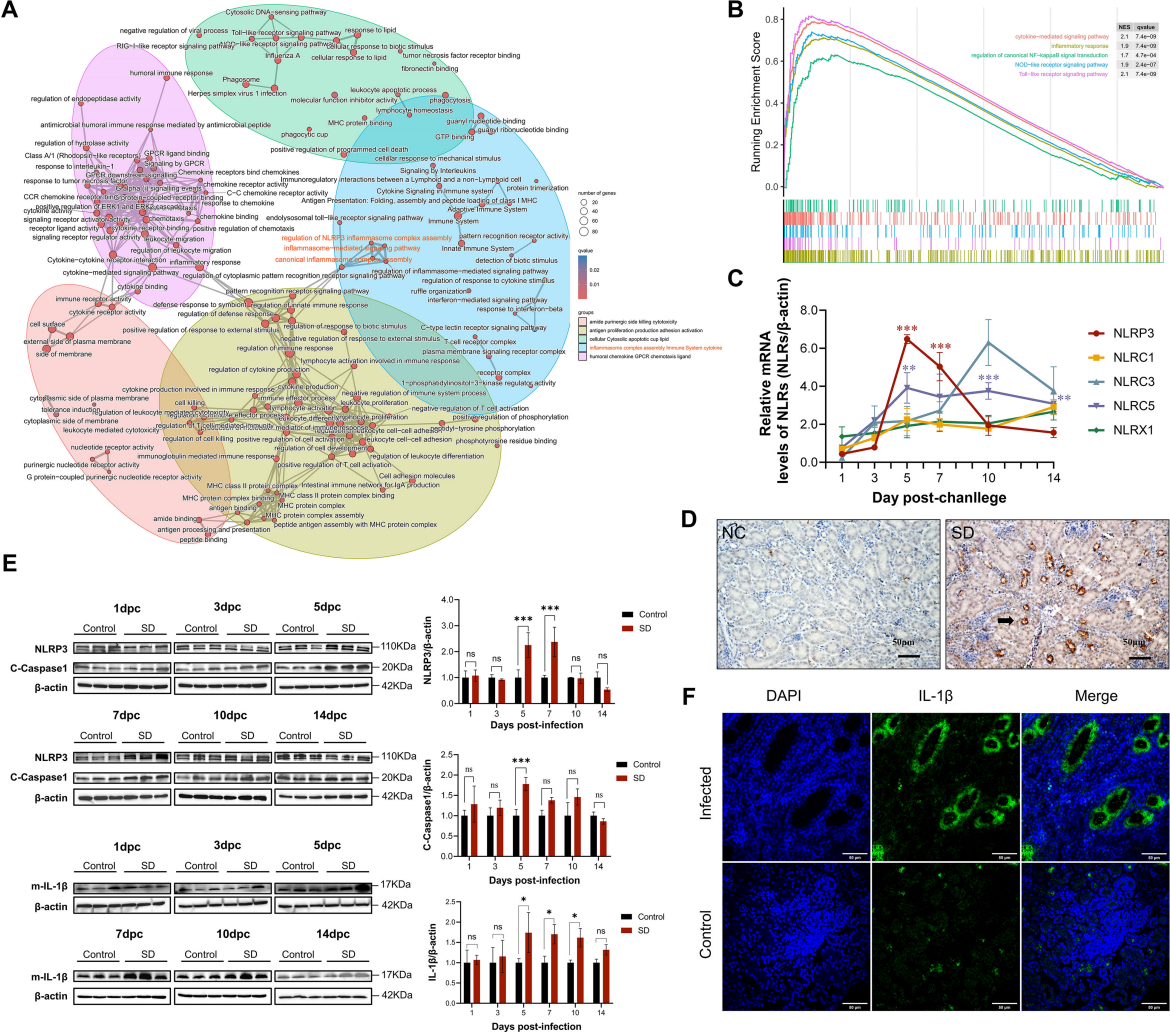
NLRP3 inflammasome activation in chicken kidneys after IBV infection. (**A**) Enrichment network of upregulated pathways in chicken kidneys following IBV infection at 5 dpi. Each node represents a significantly enriched pathway, color-coded by adjusted *P* value (*q* value), with edges indicating functional similarity among pathways. (**B**) GSEA of transcriptomic data from IBV-infected renal tissue, revealing significantly enriched pathways (*P* < 0.01). (**C**) RT-qPCR analysis of selected NLR family members’ expression in renal tissues of control and IBV-infected chickens at 1, 3, 5, 7, 10, and 14 dpi. Data were normalized to β-actin. (**D**) Immunohistochemical analysis of NLRP3 expression in renal tissue at 5 dpi. Compared to controls, IBV-infected chickens exhibited increased NLRP3-positive staining (brown; indicated by black arrows) in tubular epithelial cells. Nuclei were counterstained with hematoxylin. Scale bar = 50 µm. (**E**) Western blot analysis of NLRP3, cleaved Caspase-1, and mature IL-1β in renal tissues from control and IBV-infected chickens at the indicated time points. Representative immunoblots (left) and corresponding densitometric quantification (right) are presented. β-actin served as the loading control. Data are shown as mean ± SD (*n* = 3); statistical significance was determined by two-way ANOVA. ns, not significant; *, *P* < 0.05; **, *P* < 0.01; ***, *P* < 0.001. (**F**) Immunofluorescence staining of renal tissue revealing increased IL-1β-positive signals (green fluorescence, white arrows). Scale bar = 50 µm.

To elucidate the molecular mechanisms underlying inflammasome activation in response to IBV infection, we conducted transcriptomic profiling using RNA-seq on kidney tissues obtained from IBV-infected chickens. Differential gene expression analysis was subsequently performed to identify host genes that were significantly modulated during infection. Pathway enrichment analysis of these differentially expressed genes (DEGs) indicated a significant activation of signaling cascades associated with the NLRP3 inflammasome. Specifically, enrichment was observed in pathways involved in inflammasome complex assembly, upstream signal transduction events, and canonical inflammasome activation processes. Taken together, these findings indicate the involvement of NLRP3-mediated signaling during IBV pathogenesis (Fig. 2A).

In parallel, gene set enrichment analysis (GSEA) further corroborated these findings, revealing significant enrichment of gene sets related to inflammasome activation, NOD-like receptor signaling, Toll-like receptor signaling, cytokine-mediated communication, and general inflammatory responses (Fig. 2B). These transcriptomic results provide compelling evidence that IBV infection in chickens leads to the activation of the NLRP3 inflammasome in renal tissues. Moreover, the data suggest that IBV triggers a widespread activation of innate immune signaling pathways, highlighting a potential mechanistic link between viral infection and host inflammatory responses.

We next performed qRT-PCR analysis to assess the expression levels of key NLRs in infected kidney tissues, aiming to clarify the molecular basis of IBV-induced inflammasome activation. Among the NLR family members analyzed, NLRP3 mRNA expression was the most significantly upregulated following IBV infection, indicating that NLRP3 is likely the primary inflammasome sensor involved in the renal immune response to IBV (Fig. 2C; Fig. S1). To validate these findings at the protein level and determine the spatial distribution of NLRP3 expression, immunohistochemistry (IHC) was performed on kidney sections from infected chickens. Enhanced NLRP3 immunoreactivity was observed in distinct renal tubular structures, indicative of spatially restricted activation of the inflammasome within renal tubular compartments (Fig. 2D).

Western blot (WB) further confirmed inflammasome activation in infected kidneys relative to uninfected controls, showing significant increases in the protein levels of NLRP3, cleaved Caspase-1, and mature IL-1β at 5 and 7 dpi (Fig. 2E). In agreement with protein-level findings, immunohistochemical analysis at 5 dpi showed markedly increased IL-1β staining in kidney tissues, consistent with enhanced IL-1β expression in infected kidney tissue (Fig. 2F). IHC and immunofluorescence analyses were performed on kidney samples collected at 5 dpi, a time point selected based on peak histopathological changes observed in previous experiments.

These results indicate that NLRP3 is involved in the renal immune response to IBV infection, likely through the activation of canonical inflammasome pathways and subsequent IL-1β maturation and secretion. These findings suggest that NLRP3 may act as an upstream regulator of renal inflammation during IBV infection.

### IBV infection activates the NLRP3–Caspase-1–IL-1β pathway in CEK cells

**Fig 3:**
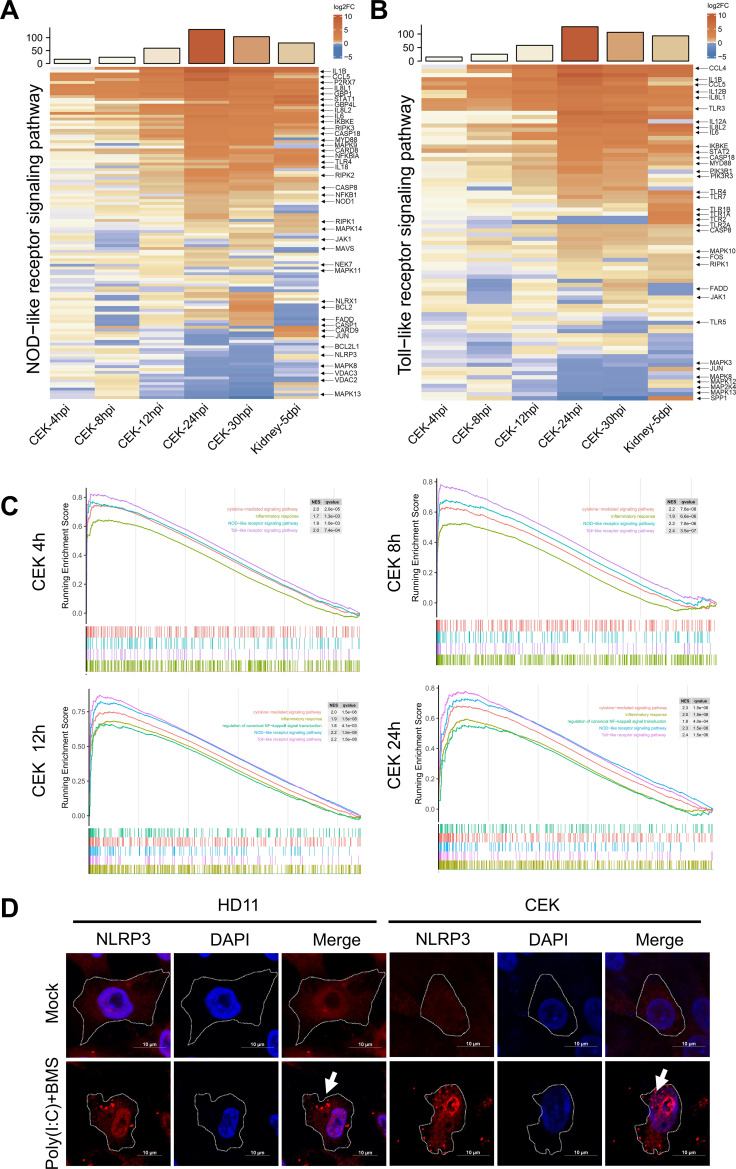
Transcriptomic analysis of IBV-infected kidney tissue and CEK cells and chemically induced NLRP3 puncta formation in HD11 and CEK cells. (**A and B**) Heatmaps show the differential expression of pattern recognition receptor (PRR) genes in CEK cells at 4, 8, 12, 24, and 30 hpi with IBV, and in kidney tissue at 5 dpi, relative to uninfected controls. Expression values are shown as log_2_ fold changes. (**C**) GSEA of transcriptomic data from IBV-infected CEK cells at 4, 8, 12, and 24 hpi identified multiple significantly upregulated pathways (*P* < 0.01). (**D**) Immunofluorescence staining of CEK and HD11 cells co-treated with Poly(I:C) (10 µg/mL, 4 h) and BMS (0.2 µM, 6 h) shows punctate NLRP3 localization (white arrows). Scale bar = 10 µm.

Transcriptomic analysis of IBV-infected primary CEK cells demonstrated a marked enrichment of signaling pathways associated with inflammasome activation, innate immune recognition, and proinflammatory responses (Fig. 3A through C). These transcriptional alterations are consistent with the immune signatures observed in renal tissues from IBV-infected chickens, thereby providing consistent evidence of inflammasome involvement in host antiviral defense. Notably, inflammasome activation is characterized by NLRP3 oligomerization and the formation of punctate cytoplasmic aggregates, which represent hallmark features of inflammasome assembly (17, 18). To establish an experimental reference for NLRP3 activation, positive control conditions were generated in CEK and HD11 cells using a classical two-signal priming protocol, in which cells were first exposed to Poly(I:C), followed by stimulation with the potassium ionophore BMS. This sequential treatment reliably induced the formation of punctate NLRP3 aggregates (Fig. 3D), thereby validating the assay system.

**Fig 4:**
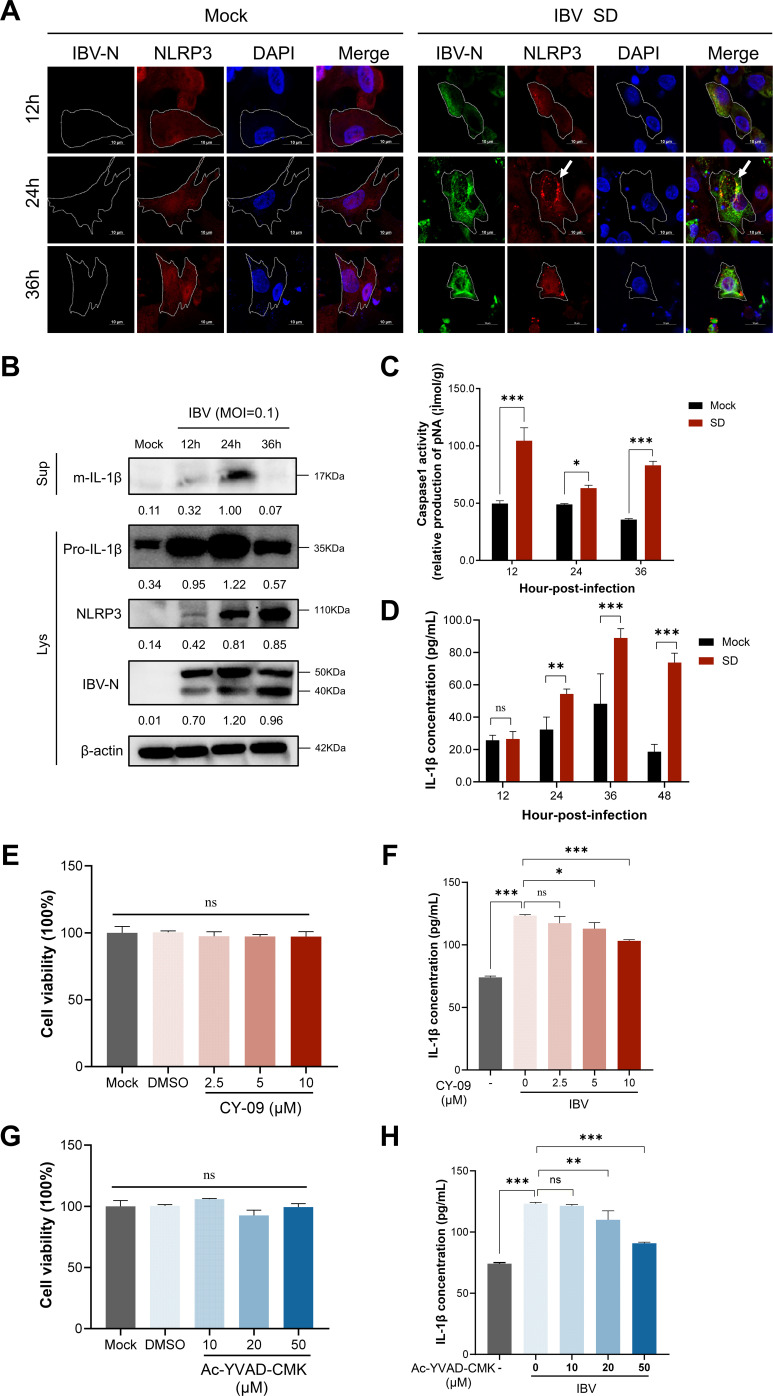
NLRP3 puncta formation and IL-1β secretion in IBV-infected CEK cells are NLRP3/Caspase-1–dependent. (**A**) Immunofluorescence analysis of NLRP3 localization in CEK cells infected with IBV (MOI = 0.1) and fixed at 12, 24, and 36 hpi. Confocal microscopy revealed redistribution of NLRP3 into punctate aggregates (white arrows). Scale bar = 10 µm. (**B**) WB analysis of NLRP3 inflammasome-related proteins in cell lysates and culture supernatants from IBV-infected CEK cells (MOI = 0.1) at 24 hpi. β-actin served as the loading control. (**C and D**) Caspase-1 activity (**C**) and IL-1β secretion (**D**) were assessed by assay kit and ELISA, respectively, after IBV infection. (**E–H**) CCK-8 assay of CEK cell viability after 24-hour treatment with graded concentrations of CY-09 (**E**) or Ac-YVAD-CMK (**G**). IL-1β secretion following NLRP3 (**F**) or Caspase-1 (**H**) inhibition in IBV-infected CEK cells. Cells were pretreated with CY-09 or Ac-YVAD-CMK for 2 h, infected with IBV (MOI = 0.1), and maintained in inhibitor-containing medium for 24 h. IL-1β levels in supernatants were quantified by ELISA. All data are representative of at least three independent experiments and are presented as mean ± SD, *n* = 3. ns, not significant; *, *P* < 0.05; **, *P* < 0.01; ***, *P* < 0.001.

The subcellular distribution of NLRP3 during IBV infection was further examined by immunofluorescence microscopy. Co-staining of NLRP3 with the IBV nucleocapsid (N) protein, a marker of productive infection, revealed a striking redistribution of NLRP3 from a diffuse cytoplasmic pattern in uninfected cells to concentrated perinuclear puncta in IBV-N-positive cells (Fig. 4A). This relocalization is indicative of inflammasome complex assembly within the infected host cell cytoplasm.

These microscopic findings were validated by immunoblotting and enzymatic activity measurements. At 24 hours post-infection (hpi), western blot analysis revealed marked upregulation of NLRP3 together with increased expression of pro-IL-1β in CEK cells, which was accompanied by enhanced secretion of mature IL-1β into the culture supernatant (Fig. 4B). Consistently, Caspase-1 enzymatic activity assays demonstrated significant activation of Caspase-1 following IBV infection, accompanied by elevated IL-1β release (Fig. 4C and D). To further confirm the roles of NLRP3 and Caspase-1 in IBV-induced IL-1β secretion, CEK cells were treated with the NLRP3 inhibitor CY-09 and the Caspase-1 inhibitor Ac-YVAD-CMK. Both inhibitors significantly reduced IL-1β secretion without affecting CEK cell viability (Fig. 4E through H). These findings together indicate that IBV infection activates the NLRP3 inflammasome in CEK cells through a canonical NLRP3–Caspase-1 axis, leading to IL-1β maturation and release, and underscore the central role of inflammasome signaling in the innate immune response to IBV infection. To assess strain dependence, CEK cells were infected with the M41 strain, which is confined to the respiratory tract. Western blot analysis showed no increase in NLRP3 expression, indicating that M41 does not activate the NLRP3 inflammasome (Fig. S2A).

### NLRP3 mediates IBV-induced renal injury

**Fig 5:**
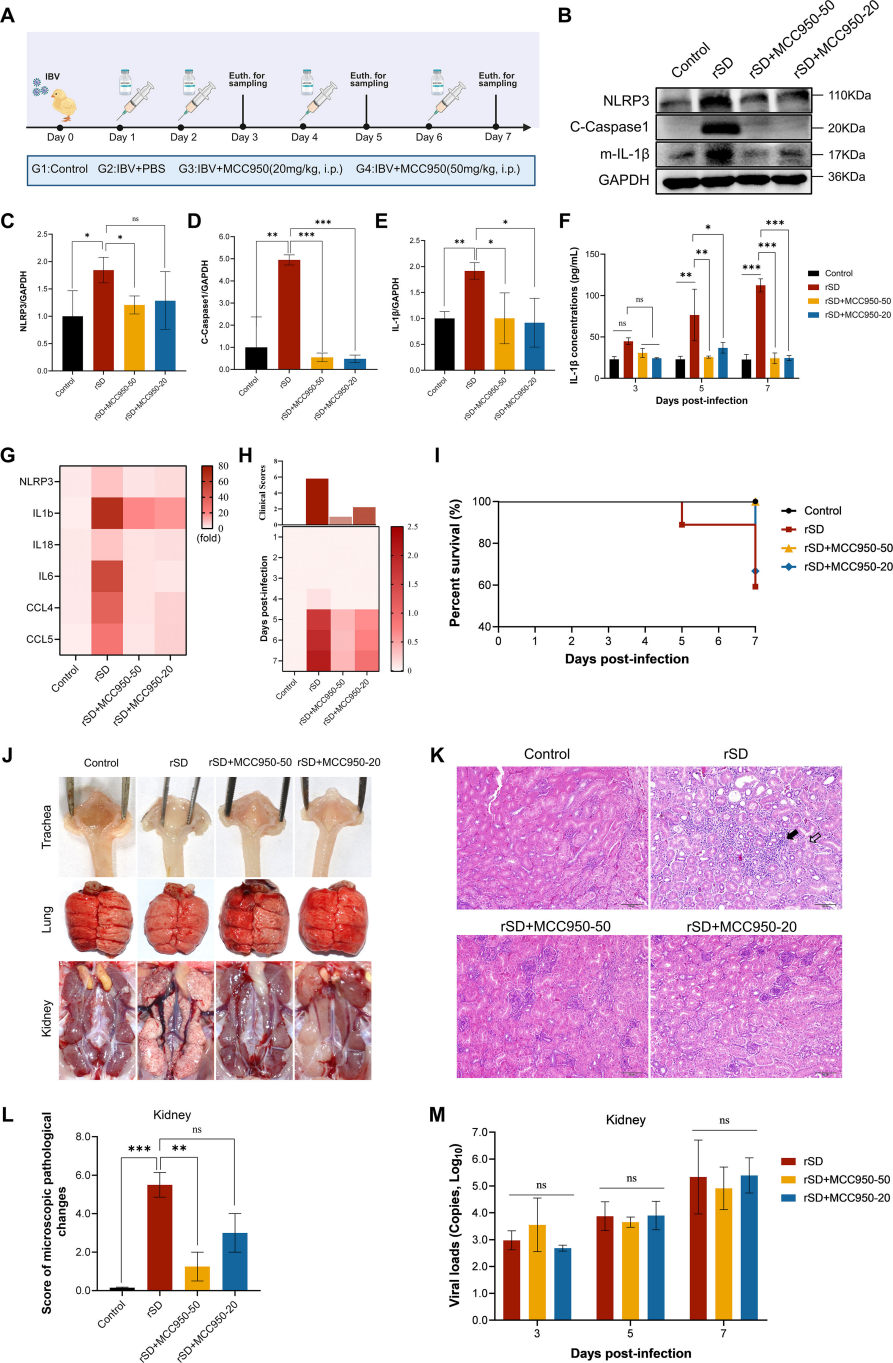
Effects of MCC950 on inflammasome activation and renal injury following IBV infection. (**A**) Schematic of *in vivo* design showing MCC950 treatment after IBV infection. Created with BioRender.com. (**B–E**) WB analysis of NLRP3, cleaved Caspase-1, and mature IL-1β in renal tissues at 5 dpi. (**B**) Representative immunoblots. (**C–E**) Quantification of the indicated proteins. β-actin served as a loading control. (**F**) Quantification of serum IL-1β levels by ELISA at 5 dpi. (**G**) qRT-PCR analysis of NLRP3, IL1B, IL18, IL6, CCL4, and CCL5 expression in renal tissues of each group. Expression was normalized to β-actin. (**H**) Clinical symptom scores were recorded daily and visualized as a heatmap. Clinical signs were scored as follows: 0 (normal), 1 (mild nasal discharge, slight tremors, and tearing), 2 (depressive behavior, watery diarrhea, coughing, or sneezing), 3 (severe nasal discharge, marked depression, open-mouth breathing, or tracheal rales), and 4 (death). (**I**) Survival curve depicting the percentage survival in each group over a 7-day observation period. (**J**) Gross pathology of the trachea, lungs, and kidneys on 5 dpi across experimental groups. (**K**) Histopathological changes in renal tissues by H&E staining. Filled arrows indicate interstitial inflammatory infiltration; open arrows show vacuolar degeneration and tubular epithelial necrosis. These changes were prominent in the IBV group and markedly reduced by MCC950. Scale bar = 50  µm. (**L**) The microscopic pathological changes were scored as follows: 0 (no microscopic lesions), 1–3 (mild lesions), 4–6 (moderate lesions), and 7–10 (severe and extensive lesions). (**M**) Viral loads in the kidney determined by qRT-PCR targeting viral RNA. All data are presented as mean ± SD, *n* = 3. ns, not significant; *, *P* < 0.05; **, *P* < 0.01; ***, *P* < 0.001.

To assess the contribution of the NLRP3 inflammasome to IBV-induced renal injury, chickens infected with IBV were treated with the specific NLRP3 inhibitor MCC950 (Fig. 5A). At 5 dpi, MCC950 treatment markedly decreased renal expression of NLRP3, cleaved Caspase-1, and mature IL-1β proteins compared with IBV-infected control chickens (Fig. 5B through E). Consistently, ELISA analysis of serum samples showed significantly lower IL-1β concentrations in MCC950-treated groups (Fig. 5F). In addition, qRT-PCR analysis revealed that MCC950 treatment substantially reduced the transcription of multiple inflammasome-associated cytokines and chemokines in renal tissues (Fig. 5G). These findings demonstrate that MCC950 effectively suppresses NLRP3 inflammasome activation and attenuates downstream proinflammatory responses triggered by IBV infection.

*In vivo* inhibition of NLRP3 by MCC950 markedly attenuated disease progression, as evidenced by decreased clinical scores and reduced mortality in infected chickens (Fig. 5H and I). Gross pathological examination revealed lesions in the trachea of both IBV-infected and MCC950-treated groups, whereas no conspicuous macroscopic changes were observed in the lungs of any group. The major gross differences were renal, with MCC950 treatment reducing mottling, swelling, and urate deposition (Fig. 5J). Histopathological analysis further confirmed that MCC950 treatment alleviated microscopic lesions, including extensive inflammatory cell infiltration and tubular necrosis (Fig. 5K and L). Importantly, these protective effects occurred without significant changes in renal viral load (Fig. 5M), indicating that the protective efficacy of MCC950 is mainly attributable to modulation of host inflammatory responses rather than direct effects on viral replication. MCC950 treatment failed to alleviate IBV-induced tracheal ciliostasis in infected chickens (Fig. S3A). Similarly, histopathological and viral load analyses showed no improvement in tracheal or lung lesions compared with the IBV-infected group (Fig. S3B through D). Taken together, these findings indicate a central role of NLRP3 inflammasome activation in IBV-induced kidney injury and point to NLRP3 inhibition as a potential therapeutic approach to mitigate renal pathology and mortality in infected chickens.

### Collecting duct-specific NLRP3 inflammasome activation by IBV leads to inflammation and uric acid accumulation

**Fig 6:**
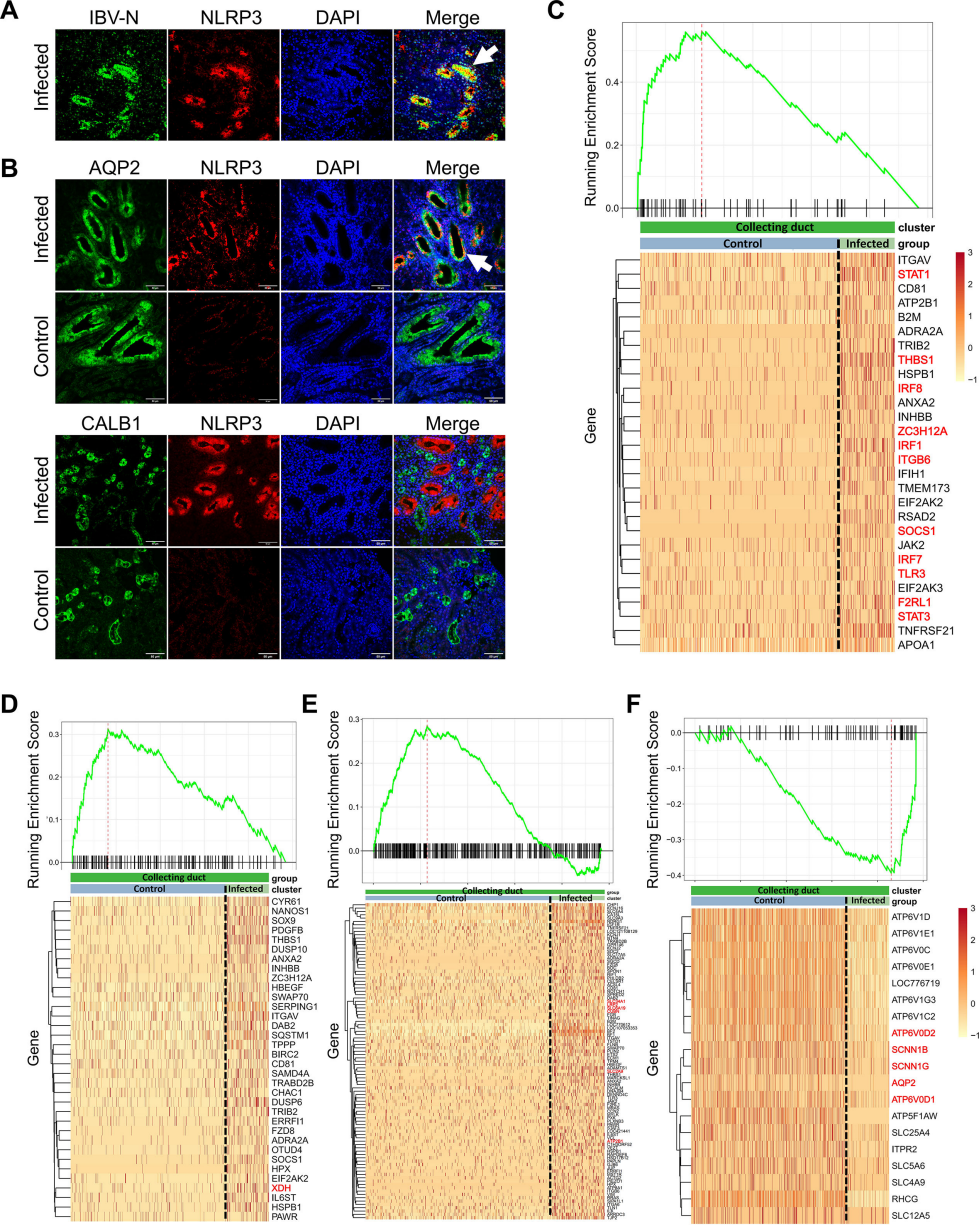
IBV-induced NLRP3 inflammasome activation and impaired ion transport in collecting duct epithelial cells. (**A**) Dual immunofluorescence staining of paraffin-embedded chicken kidney sections at 5 dpi with IBV, followed by confocal microscopy to detect co-localization of IBV-N and NLRP3 in tubular epithelial cells. (**B**) Dual IFA of IBV-infected kidney tissue showing NLRP3 expression in AQP2-positive collecting duct cells, but not in CALB1-positive distal tubule cells. (**C–F**) Single-cell RNA sequencing (scRNA-seq) analysis of collecting duct cells isolated from chicken kidneys at 5 dpi. The panels include GSEA plots and heatmaps showing DEGs between IBV-infected and control groups. Upper panels present GSEA results showing significantly enriched or suppressed pathways. Lower panels display heatmaps of representative pathway-associated genes across individual cells from control (left of dashed line) and infected (right of dashed line) samples. Color intensity reflects relative gene expression levels.

Our earlier work provided evidence that IBV infects both distal tubular and collecting duct epithelial cells in the chicken kidney (19). To explore IBV-induced renal inflammation, kidney tissues collected at 5 dpi were analyzed by immunofluorescence for IBV nucleocapsid (N) and NLRP3 proteins. NLRP3 expression was upregulated and colocalized with IBV-N in specific renal tubules (Fig. 6A), indicating inflammasome activation in tubular epithelial cells. Dual staining with AQP2 and CALB1 showed NLRP3 upregulation predominantly in AQP2 (Aquaporin-2)-positive collecting duct cells, but not in CALB1 (Calbindin)-positive distal tubules (Fig. 6B), that NLRP3 activation occurs predominantly in collecting duct epithelia. In contrast, qPCR and immunofluorescence analyses (IFAs) showed no increase in NLRP3 expression in tracheal tissues after IBV infection, indicating that NLRP3 inflammasome activation does not occur in the trachea (Fig. S2B and C).

To clarify IBV-induced inflammation’s role in kidney injury, single-cell RNA sequencing (scRNA-seq) of collecting duct cells revealed a strong innate immune response. GSEA showed upregulation of viral sensors (*TLR3*, *IFIH1*, and *EIF2AK2*) and downstream pathways (IRF signaling, *JAK*/*STAT*, *AP-1*; Fig. S4A and B). Key transcription factors (*STAT1/3*, *IRF1/7/8*) were elevated, likely driving pro-inflammatory cytokine and chemokine expression (Fig. 6C). Immune regulatory and adhesion molecules (*IL1RAP*, *F2RL1*, and *THBS1-ITGB6*) were differentially expressed, suggesting enhanced immune cell recruitment and inflammation via altered cell interactions (Fig. S4B). Notably, negative regulators (*SOCS1*, *DUSP10*, and *ZC3H12A*) were also upregulated, indicating feedback to limit inflammation (Fig. 6C; Fig. S4C).

In chickens, uric acid is the primary nitrogenous waste excreted mainly via collecting ducts as urate salts (20, 21). Our data show that IBV infection disrupts urate metabolism and transport in collecting duct cells. GSEA revealed upregulation of purine metabolism and the rate-limiting enzyme xanthine dehydrogenase (*XDH*) (Fig. 6D), indicating increased uric acid synthesis and accumulation. Simultaneously, membrane transport pathways were activated, including elevated expression of the urate transporter SLC2A9 (*GLUT9*; Fig. 6E), suggesting enhanced tubular urate reabsorption contributing to renal urate buildup.

Conversely, ion and small-molecule transport pathways crucial for cellular homeostasis were significantly downregulated. GSEA showed suppression of ion transport, small molecule metabolism, and V-ATPase complex pathways vital for tubular acidification and urine pH regulation. Key transport genes, including *AQP2*, *SCNN1G*, *SCNN1B*, and V-ATPase subunits *ATP6V0D1* and *ATP6V0D2*, were markedly decreased (Fig. 6F; Fig. S4D and E). In chickens, impaired tubular regulation of water, ions, and acid-base balance may reduce urate solubility and facilitate crystal formation during hyperuricemic states.

In conclusion, IBV infection induces a pathological state in collecting duct epithelia characterized by persistent inflammasome activation, altered uric acid metabolism, and impaired electrolyte and acid-base transport. These dysfunctions create a pro-inflammatory, metabolically imbalanced microenvironment that promotes urate crystal formation and hinders renal excretion. Urate deposition further amplifies inflammation via a positive feedback loop, contributing to progressive kidney injury and increased mortality in infected chickens.

## DISCUSSION

IBV-induced renal pathology is strain dependent. Mutations in the S1 subunit of the spike glycoprotein largely determine kidney tropism by modulating receptor binding and viral entry (22). Nephropathogenic lineages (e.g., GI-19 and GI-7) are associated with severe renal lesions (3), while accessory and nonstructural proteins further influence virulence and host immune responses (23, 24).

Proinflammatory cytokines are key mediators of the innate immune response, initiating and modulating inflammation during viral infection (25, 26). A range of viral pathogens can upregulate the expression of proinflammatory cytokines, such as interleukin (IL)−1β, IL-18, and IL-8, thereby activating inflammatory signaling pathways, promoting immune cell recruitment, and potentially causing tissue damage (27, 28). Viruses such as SARS-CoV-2, MERS-CoV, and influenza virus have been reported to induce marked pathological changes in host tissues by promoting excessive cytokine production (13, 14, 29). In this study, GI-19 genotype IBV infection in chickens induced a marked inflammatory response in renal tissues. The major pathological features included multifocal mottled renal lesions, disruption of tubular architecture, and extensive infiltration of inflammatory cells. Consistently, the expression of *IL1B*, *IL18*, and *IL8* mRNA was significantly upregulated in renal tissues from infected chickens, further supporting the role of GI-19 IBV infection in mediating renal inflammation.

The production of proinflammatory cytokines is largely mediated by inflammasome activation (30). Inflammasomes, as cytosolic multiprotein complexes, orchestrate pathogen-induced inflammatory signaling through caspase-1 activation, leading to the maturation and secretion of IL-1β and IL-18 (31). Multiple studies have demonstrated that viruses such as dengue virus, IAV, and enterovirus 71 (EV71) activate the NLRP3 inflammasome (32–34). NLRP3 activation is a key feature of coronavirus infections. It contributes to cytokine storms in severe SARS-CoV-2 infection, promotes inflammation and tissue damage during MERS-CoV infection, and drives inflammatory responses in SARS-CoV (35). However, whether the infectious bronchitis virus activates the NLRP3 inflammasome to induce inflammation and thereby contribute to tissue damage remains unclear. In this study, we demonstrate that IBV activates the NLRP3 inflammasome both *in vivo* and *in vitro*, thereby promoting the maturation and release of proinflammatory cytokines through the NLRP3–caspase-1–IL-1β signaling axis. To our knowledge, this is the first study to demonstrate that IBV infection activates the NLRP3 inflammasome in animal models, leading to IL-1β secretion and subsequent inflammatory responses. Similar to SARS-CoV-2 and MERS-CoV, where NLRP3 activation is triggered by mitochondrial ROS, K^+^ efflux, and lysosomal destabilization (36), viral proteins such as ORF3a, E, and N can further enhance activation via ion flux or interaction with inflammasome components (37). IBV may employ comparable pathways, and elucidating these mechanisms will advance our understanding of coronavirus-induced inflammasome regulation. We confirmed the activation of the NLRP3 inflammasome, underscoring its key role in IBV-induced renal inflammation. To further assess this, we used MCC950, a selective inhibitor of NLRP3 assembly and Caspase-1 activation via ATPase inhibition. Mechanistically, MCC950 directly engages the NACHT domain of NLRP3—at or near the Walker-B/ATPase site—thereby blocking ATP hydrolysis and locking NLRP3 in a closed, inactive conformation that prevents oligomerization and ASC speck formation (38). MCC950 specifically inhibits NLRP3 without affecting other inflammasomes (i.e., it does not inhibit AIM2, NLRC4, or NLRP1 under conditions where NLRP3 activation is potently suppressed) (39) and has shown efficacy in reducing inflammation and improving survival in SARS-CoV-2 models (40). In this study, MCC950 treatment in IBV-infected chickens did not alter renal viral load but significantly reduced the protein levels of NLRP3, cleaved Caspase-1, IL-1β, serum proinflammatory cytokines, and chemokine mRNA levels. Histopathology showed marked attenuation of renal inflammation, accompanied by improved clinical signs and survival. These results indicate that NLRP3 hyperactivation drives renal inflammation and pathology in IBV infection, and MCC950 provides protection by targeting this pathway without affecting viral replication.

Although NLRP3 plays a critical role in renal inflammation, MCC950 treatment failed to mitigate IBV-induced respiratory damage, including tracheal ciliostasis and lung lesions, and did not significantly reduce viral loads in these tissues (Fig. S3). These findings suggest that NLRP3 contributes to IBV pathogenesis in a tissue-specific manner, being more important in kidney injury than in respiratory impairment. This study demonstrates that in CEK cells, IBV infection activates the NLRP3 inflammasome cascade, promoting maturation and release of IL-1β. Notably, avian and mammalian NLRP3 inflammasomes differ significantly in molecular architecture and activation mechanisms (41). In mammals, the activation of NLRP3 involves protein oligomerization, a process that facilitates the recruitment of ASC and pro-Caspase-1, ultimately triggering inflammasome assembly (42). ASC is typically considered a morphological marker of NLRP3 assembly (43). Previous studies have reported that chickens, unlike mammals, lack the ASC-encoding gene (35). This evolutionary divergence leads to the absence of canonical ASC specks, posing challenges for elucidating the mechanisms of IBV-induced NLRP3 activation in avian models. Although ASC is absent, previous studies have demonstrated that chicken NLRP3 can mediate IL-1β maturation and secretion, indicating a potentially ASC-independent mechanism involving oligomerization-driven recruitment of downstream signaling components (44, 45). Supporting this, treatment with the NLRP3 inhibitor CY-09 or Caspase-1 inhibitor Ac-YVAD-CMK significantly reduced IL-1β secretion, implicating the NLRP3/Caspase-1 axis. Thus, the perinuclear punctate NLRP3 aggregates observed in chicken cells may serve as alternative morphological markers of inflammasome activation in the absence of ASC.

Previous studies on the NLRP3 inflammasome have focused primarily on immune cells such as macrophages (46), but recent evidence highlights the role of epithelial cells in pathogen sensing and inflammation (47). In renal disease, tubular epithelial cells have been identified as key targets of NLRP3 activation (48). Experimental data show that CEK cells activate the NLRP3 inflammasome upon IBV infection, suggesting that renal epithelial cells are both targets of infection and active contributors to inflammation. *In vitro* models further reveal species-specific differences in avian inflammatory signaling compared to mammals. In mammalian cells, canonical NLRP3 activation typically follows a two-signal model: a priming step (Signal 1, e.g., LPS) and an activation step (Signal 2, e.g., nigericin) (8). However, this dual-signal protocol fails to activate NLRP3 in CEK cells, likely due to inefficient lipopolysaccharide (LPS) sensing via chicken TLR4 and attenuated TRIF-dependent signaling, leading to weak NF-κB activation and insufficient NLRP3 priming (49, 50). Stimulation with the TLR3 agonist Poly(I:C) (51) and the small-molecule NLRP3 activator BMS (46) induced NLRP3 puncta in CEK cells, but western blotting showed no significant increase in NLRP3 protein levels. These findings suggest that the current stimulation protocols may be inadequate for robust NLRP3 activation in CEK cells, representing a limitation of this study.

While human coronaviruses, including SARS-CoV-2, are known to activate the NLRP3 inflammasome in renal tissues and contribute to acute kidney injury, the spatial and temporal dynamics of this activation in specific tubular epithelial subtypes remain poorly understood (52–54). In this study, dual immunofluorescence staining revealed that IBV-induced NLRP3 expression is predominantly localized to collecting duct epithelial cells in chickens—a previously unreported anatomic distribution, pointing to the collecting duct as a potential site of virus-driven inflammation. Given its essential role in water and electrolyte homeostasis, collecting duct dysfunction may destabilize renal physiology (55). Our data show that IBV infection induces a pro-inflammatory phenotypic shift in collecting duct epithelial cells, implicating this segment as a key contributor to renal inflammation. Notably, chickens excrete nitrogen as uric acid, primarily via urate crystals in the collecting ducts (20, 21), making them especially vulnerable to urate-related injury. IBV infection upregulates *XDH* and *SLC2A9*, increasing uric acid production and reabsorption, which raises uric acid levels and promotes urate crystal formation in the collecting ducts. These crystals act as damage-associated molecular patterns (DAMPs) to activate the NLRP3 inflammasome and amplify inflammation (56). Our findings suggest that IBV triggers a “urate crystal–inflammation” feedback loop that drives renal injury progression, akin to gout-associated nephropathy in humans (57, 58). Thus, IBV-induced NLRP3 activation in collecting duct epithelial cells establishes a pathogenic link between urate metabolism disruption and inflammation, offering new insights into IBV-related renal pathogenesis.

## MATERIALS AND METHODS

### Viruses and cells

The SD and M41 subtype of IBV used in this study was previously isolated and identified by our team. HD11 cells were maintained in RPMI 1640 medium (Gibco, USA) supplemented with 10% fetal bovine serum (FBS) and 1% penicillin-streptomycin. CEK cells were isolated from 18-day-old SPF chicken embryos. All cells were incubated at 37°C in a humidified atmosphere containing 5% CO_2_.

### Reagents and antibodies

Chicken IL-1β ELISA kit (SEA563Ga) and IL-18 ELISA kit (SEA064Ga) were purchased from Cloud-Clone Product, China. Total RNA Isolation Kit was purchased from Magen, Beijing, China. M5 HiPer Real-Time PCR Super Mix was purchased from Mei5bio, Beijing, China. Caspase 1 Activity Assay Kit, Immunol Staining Fix Solution, Immunostaining Permeabilization Buffer containing Triton X-100, Immunol Staining Blocking Buffer, CCK-8 solution, and Cell Lysis Buffer for Western were purchased from Beyotime, China. Chemicals used included CY-09 (MedChemExpress, HY-103666), Ac-YVAD-cmk (Selleck, S9727), MCC950 Sodium (Selleck, S7809), BMS-986299 (Selleck, S9899), and polyinosinic-polycytidylic acid (poly[I:C]; Sigma, P1530). Antibodies were mouse mab IBV-N protein (HyTes, 3BN1), rabbit pab NLRP3 (ABclonal, A24297), rabbit pab IL1β (ABclonal, A16288), rabbit pab Cleaved-caspase1 (Wanlei Biotechnology, WL03450), mouse mab β-actin (ABclonal, AC004), mouse mab GAPDH (ABclonal, AC033), rabbit pab AQP2 (Abmart, PK58037), and mouse mab calb1 (Boster Bio, BM0203). Alexa Fluor 488-conjugated anti-mouse IgG (H+L; Cell Signaling Technology, 4408) and Alexa Fluor 555-conjugated anti-rabbit IgG (H+L; Cell Signaling Technology, 4413).

### Enzyme-linked immunosorbent assay

The concentrations of IL-1β and IL-18 in cell culture supernatants and serum were quantified by ELISA according to the manufacturer’s instructions. Briefly, 100  µL of standards and test samples were added to each well of a pre-coated 96-well microplate and incubated at 37°C for 1 hour. After discarding the contents, the plate was gently blotted dry on absorbent paper. Then, 100  µL of biotin-labeled anti-IL-1β antibody was added to each well and incubated at 37°C for an additional hour, followed by three washes with wash buffer. Subsequently, 100  µL of horseradish peroxidase (HRP)-conjugated streptavidin was added, and the plate was incubated at 37°C for 30 minutes, followed by five washes. Thereafter, 90  µL of TMB substrate was added to each well and incubated in the dark at 37°C for 10–20 minutes. The reaction was terminated by adding 50 µL of stop solution, and the optical density was measured at 450  nm.

### Caspase-1 activity assay

Caspase-1 activity in cell lysates was determined using a Caspase-1 Activity Assay Kit (Beyotime, China). Briefly, 50 µL of cell lysate was mixed with 40 µL of assay buffer, followed by the addition of 10 µL of Ac-YVAD-pNA (2 mM) substrate, yielding a final volume of 100 µL. For the blank control, the lysis buffer was used in place of the sample. The reaction mixture was incubated at 37°C until visible color development, and the absorbance was measured at 405 nm using a microplate reader.

### Real-time quantitative PCR

Total RNA was extracted from cells or tissues using an RNA extraction kit and reverse-transcribed to generate cDNA. The resulting cDNA was amplified by real-time quantitative PCR (RT-qPCR) using the M5 HiPer Real-Time PCR Super Mix. Thermal cycling conditions were initial denaturation at 95°C for 30 s; 40 cycles of 95°C for 5 s and 60°C for 30 s; followed by melt-curve analysis from 60°C to 90°C at a ramp rate of 1°C/s. The expression levels of target genes were normalized to β-actin mRNA expression. Detailed primer sequences are listed in Table S1.

### Detection of viral RNA in tissue samples

Total RNA was extracted from cells or tissue homogenates using a commercial RNA extraction kit with on-column DNase treatment, and first-strand cDNA was synthesized using a standard reverse-transcription kit. IBV RNA was quantified by RT-qPCR (SYBR Green chemistry, M5 HiPer Real-Time PCR Super Mix) using primers targeting the conserved 5′-UTR (forward 5′-GTTGGGCTACGTTCTCGC-3′; reverse 5′-AAGCCATGTTGTCACTGTCTAT-3′; amplicon 130 bp). Each 20 µL reaction contained 2 × M5 HiPer SYBR Premix 10 µL, forward primer 0.4 µL (10 µM; 0.2 µM final), reverse primer 0.4 µL (10 µM; 0.2 µM final), cDNA 2 µL, and nuclease-free water 7.2 µL.

### Dual immunofluorescence of kidney sections

Dual immunofluorescence staining of kidney tissues was performed using the mIHC Dual Immunofluorescence Kit (Panovue, China). Briefly, paraffin-embedded kidney tissues were sectioned at a thickness of 4 µm and mounted on slides. After deparaffinization in xylene and rehydration through a graded ethanol series, heat-induced antigen retrieval was carried out in citrate-EDTA buffer (Beyotime, China) for 10 minutes. Sections were then blocked with blocking buffer at room temperature for 1 hour, followed by incubation with the first primary antibody at 4°C overnight. The next day, sections were washed three times with TBST and further rinsed according to the kit instructions. Fluorophore-conjugated secondary antibodies were applied and incubated at room temperature for 20 minutes, followed by TSA amplification for 10 minutes, and then washed. Antibody stripping was performed using the stripping buffer provided in the kit for 15 minutes, followed by TBST washing. The second round of staining was carried out by repeating the primary antibody incubation and subsequent steps as described above. After staining, the sections were coverslipped, and fluorescence signals were acquired using a Nikon A1 confocal microscope (Nikon, Tokyo, Japan).

### Indirect IFA and confocal microscopy

Cell samples were collected at predetermined time points post-transfection or infection and fixed using Immunol Staining Fix Solution. Subsequently, the cells were permeabilized with Immunostaining Permeabilization Buffer containing Triton X-100 and blocked with Immunol Staining Blocking Buffer. The cells were then incubated with specific primary antibodies at 4°C for 12 hours. For staining, Alexa Fluor 488-conjugated anti-mouse IgG (H+L) and/or Alexa Fluor 555-conjugated anti-rabbit IgG (H+L) were added and incubated at room temperature in the dark for 1 hour. Nuclei were stained with DAPI at room temperature for 10 minutes. The cells were then washed five times with phosphate-buffered saline (PBS) containing Tween 20 (PBST), with each wash lasting 5 minutes. Finally, the cells were observed and imaged using a Nikon A1 fluorescence microscope (Nikon, Tokyo, Japan). Colocalization analysis was performed using Fiji ImageJ software. The observed correlation coefficient (R[obs]) represents the Pearson correlation coefficient calculated using ImageJ, ranging from −1 to +1, where +1 indicates a perfect positive correlation (complete colocalization), 0 indicates no correlation (random distribution), and −1 indicates a perfect negative correlation.

### Drug treatment and virus infection

CEK cells were seeded into culture plates and grown to 80%–90% confluence. Prior to infection with IBV at a multiplicity of infection (MOI) of 0.01, cells were pretreated with small-molecule inhibitors at the indicated concentrations for 2 hours. Following pretreatment, the drug-containing medium was removed, and cells were infected with IBV for 2 hours at 37°C to allow viral adsorption. After infection, the viral inoculum was discarded, and cells were washed twice with PBS. Fresh maintenance medium containing the corresponding concentrations of the inhibitors was then added, and cells were incubated for an additional 24 hours at 37°C in a 5% CO_2_ incubator. After treatment, cells or supernatants were collected for downstream analyses, including IL-1β and Caspase-1 activity assays. The inhibitors used in this study included CY-09 and Ac-YVAD-cmk, all of which were administered following the same treatment protocol. Stock solutions were prepared by dissolving each compound in dimethyl sulfoxide or sterile water. Solvent control groups were prepared using equivalent volumes of the respective solvents to account for solvent-related effects.

### *In vivo* infection experiments and sample collection

Two independent *in vivo* infection experiments were conducted. In the first experiment, 1-day-old SPF chickens were randomly assigned to two groups: the infectious bronchitis virus strain SD (IBV-SD) group and the control group. Birds in the IBV-SD group were inoculated with 10^5.5^ TCID₅₀ of IBV-SD via the intranasal and ocular routes, while the control group received an equal volume of PBS.

In the second experiment, another cohort of 1-day-old SPF chicken was randomly assigned to four groups: the IBV-SD group, IBV-SD group treated with high-dose MCC950 (50 mg/kg/day), IBV-SD group treated with low-dose MCC950 (20 mg/kg/day), and a negative control group. All virus-inoculated groups received 10^5.5^ TCID₅₀ of the corresponding viral strain via the same administration route. MCC950 was administered daily via intraperitoneal injection. The dosages of MCC950 (50 mg/kg/day and 20 mg/kg/day) were selected based on previous studies involving chickens and other animals, which demonstrated effective inhibition of NLRP3 inflammasome activation at these doses (59, 60). The intraperitoneal injection route was chosen to ensure efficient drug delivery and uniform distribution in the body.

Throughout the experimental period, all chickens were monitored daily for clinical signs, including sneezing, tracheal rales, and somnolence. On designated days post-infection, three chickens from each group were randomly selected for euthanasia and necropsy. Macroscopic lesions in the trachea, lungs, and kidneys were recorded. Tracheal ciliary activity and mean lesion scores were assessed as previously described (24). Peripheral blood and tissue samples were collected aseptically. Portions of tissue samples were fixed in 10% neutral-buffered formalin for immunohistochemical or histopathological analysis, and lesion severity was evaluated according to criteria described in previous studies (61). Remaining tissues were snap frozen in liquid nitrogen and stored at –80°C for analysis of gene transcription and protein expression levels. Blood samples were centrifuged to obtain serum, and cytokine concentrations were measured using ELISA.

### Transcriptomic analysis

CEK cells were infected with IBV at 1 MOI, and the control group was treated with PBS. Each group had three replicates. Cells were harvested at 4, 8, 12, and 24 hpi for mRNA sequencing. *In vivo* experiment kidney tissues were collected from IBV-infected and control chickens at 5 dpi, with five biological replicates per group. RNA with a poly(A) tail was extracted for subsequent transcriptomic analysis. RNA-seq libraries were prepared and sequenced by BenaGen Ltd.

Raw sequencing reads were initially processed using fastp (v0.23.2) to remove low-quality reads. Clean reads were aligned to the chicken reference genome (GRCg7b, Ensembl release 114), and transcript quantification was performed using STAR (v2.7.10a). Subsequent differential gene expression analysis between IBV-infected and control groups was performed using the R package DESeq2 (v1.40.2). The resulting gene lists were used for downstream gene enrichment analysis and GSEA.

Functional annotation of genes was performed using information from the Ensembl, KEGG, and Reactome databases. DEGs were defined as those with an absolute log_2_(fold change) >2 and a Benjamini–Hochberg adjusted *P*-value < 0.01. GO enrichment analysis of the DEGs was conducted using the R package clusterProfiler (v4.8.1). To visualize the relationships between significantly enriched GO terms (*P*-value < 0.01), a GO term similarity network was constructed, where each node represents a significantly enriched GO term and edges indicate a Jaccard similarity coefficient >0.35 between associated gene sets. To assess pathway-level changes, GSEA was performed on the entire ranked list of genes using clusterProfiler. Genes within these pathways that exhibited significant differential expression (*P*-value < 0.01) at any time point were visualized as a heatmap using the R package ComplexHeatmap (v2.16.0).

### Single-cell transcriptomic analysis

We utilized a processed scRNA-seq data set from our prior work, which profiled kidney tissues from IBV-infected and control chickens (Genome Sequence Archive of National Genomics Data Center, CRA015620). The initial data processing, including quality control, normalization, dimensionality reduction, clustering, and cell type annotation, was performed as previously described (19). For the present study, we subset the collecting duct (CD) cell population, identified based on the expression of the marker gene AQP2. We then performed a differential gene expression analysis between the IBV-infected and control CD cells within the Seurat R package (v4.3.0). To identify biological pathways altered by IBV infection, we performed GSEA. All detected genes were pre-ranked based on their log_2_ fold-change values derived from the differential expression analysis. This ranked gene list was then analyzed using the gseGO function from the clusterProfiler R package (v4.6.0) with the GO database. Pathways with a *P*-value < 0.05 were considered significantly enriched. The expression patterns of leading-edge genes from significant pathways were visualized with a heatmap generated by the pheatmap package.

### Statistical analysis

All data were analyzed using GraphPad Prism (version 9.0; GraphPad Software Inc., San Diego, CA, USA). Comparisons between two groups were performed using Student’s *t*-test, and differences among multiple groups were assessed using one- or two-way analysis of variance (ANOVA). All experiments were independently repeated at least three times. Quantitative data are expressed as mean ± SD. *P* > 0.05 stands for not significant (ns); *, *P* < 0.05; **, *P* < 0.01; ***, *P* < 0.001 indicated in the figure captions.

## Data Availability

All relevant data are provided as figures within the paper and its supplemental material. Sequencing data have been deposited in the Genome Sequence Archive (GSA) of the National Genomics Data Center under the accession number CRA027401.

## SUPPLEMENTAL MATERIAL

Supplemental material jvi.01466-25-s0001.pdf Table S1; Fig. S1 to S4.

ASM does not own the copyrights to Supplemental Material that may be linked to, or accessed through, an article. The authors have granted ASM a non-exclusive, world-wide license to publish the Supplemental Material files. Please contact the corresponding author directly for reuse.
