# Supplementary material for: Avian coronavirus IBV-induced activation of the NLRP3–Caspase-1–IL-1β axis in renal collecting ducts contributes to nephropathogenesis
Source: J Virol. 2025 Dec 3;99(12):e01466-25. doi: 10.1128/jvi.01466-25 (PMC12724324; doi:10.1128/jvi.01466-25)
Supplement: Supplemental material — Table S1; Fig. S1 to S4. [file jvi.01466-25-s0001.pdf]

Supplementary Material

TABLE S1 Primers used in the study

Primer Name	Sequences (5'-3')
NLRP3-F	CGTGTTGGGCAGTTTCACAG
NLRP3-R	GCCCACTGCTTGATGGAGAA
NLRC1-F	CAGCCATTAAAGAAGTCTGTTTGA
NLRC1-R	CAGTCCGAGCGTGGC
NLRC3-F	GCCTCTGATGGAAGAGGCTT
NLRC3-R	CAGCCAGGATGTCCACCAC
NLRC5-F	CACGCTCTACACAAGAAGGGAT
NLRC5-R	AGTGGCAGAGTTTCAGCACA
NLRX1-F	TGCAGGAAGTCACCGCC
NLRX1-R	GACATCTTCTGCCCGGCTC
IL1b-F	CCTCCAGCCAGAAAGTGAGG
IL1b-R	TTGTAGCCCTTGATGCCCAG
IL18-F	AAGCGTGGCAGCTTTTGAAG
IL18-R	CTGAAGGTGCGGTGGTTTTG
IL8-F	AGATGTGAAGCTGACGCCAA
IL8-R	GAGCTGAGCCTTGGCCATAA
IL6-F	CTCGTCCGGAACAACCTCAA
IL6-R	TCAGGCATTTCTCCTCGTCG
CCL4-F	CAAAGCCTGCCATCATCTTCAT
CCL4-R	TGACGCTCTGCAGGTATCTCT
CCL5-F	TCTTCATCTCCGTTTGGGGC
CCL5-R	TCACATGATTCTGGGGCAGC

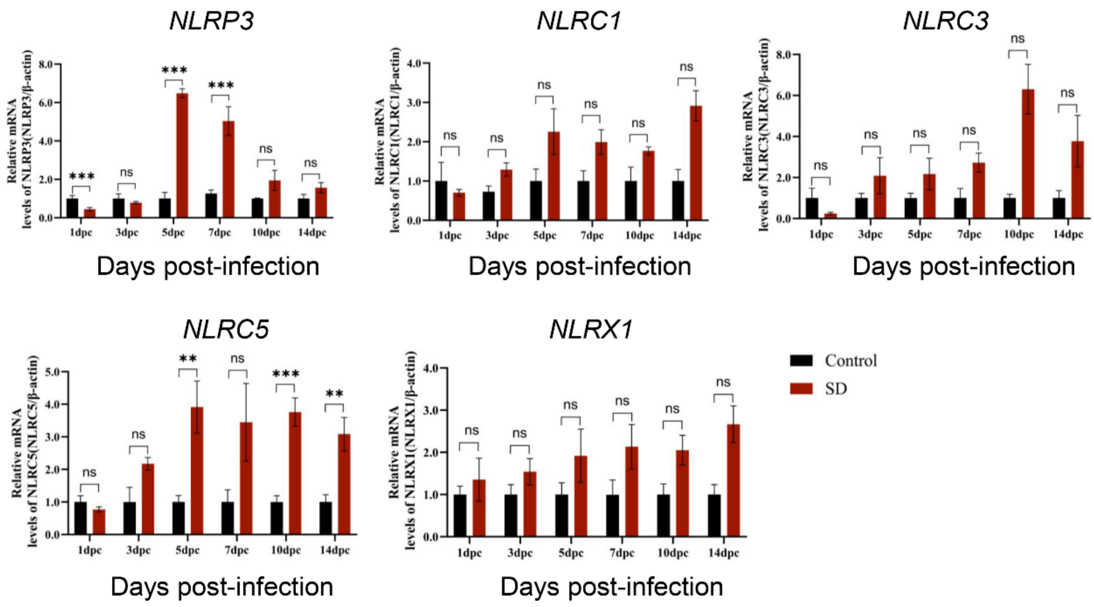

FIG S1 Time-course analysis of NLR gene expression in chicken kidneys following IBV infection. Relative mRNA levels of selected NLR family members were quantified by RT-qPCR in kidney samples collected from control and IBV-infected chickens at 1, 3, 5, 7, 10, and 14 dpi. Expression was normalized to β-actin. Data are presented as mean ±SD, n = 3. Statistical significance was assessed using two-way ANOVA. Statistical notations: ns, not significant; *, $P < 0.05$; **, $P < 0.01$; ***, $P < 0.001$.

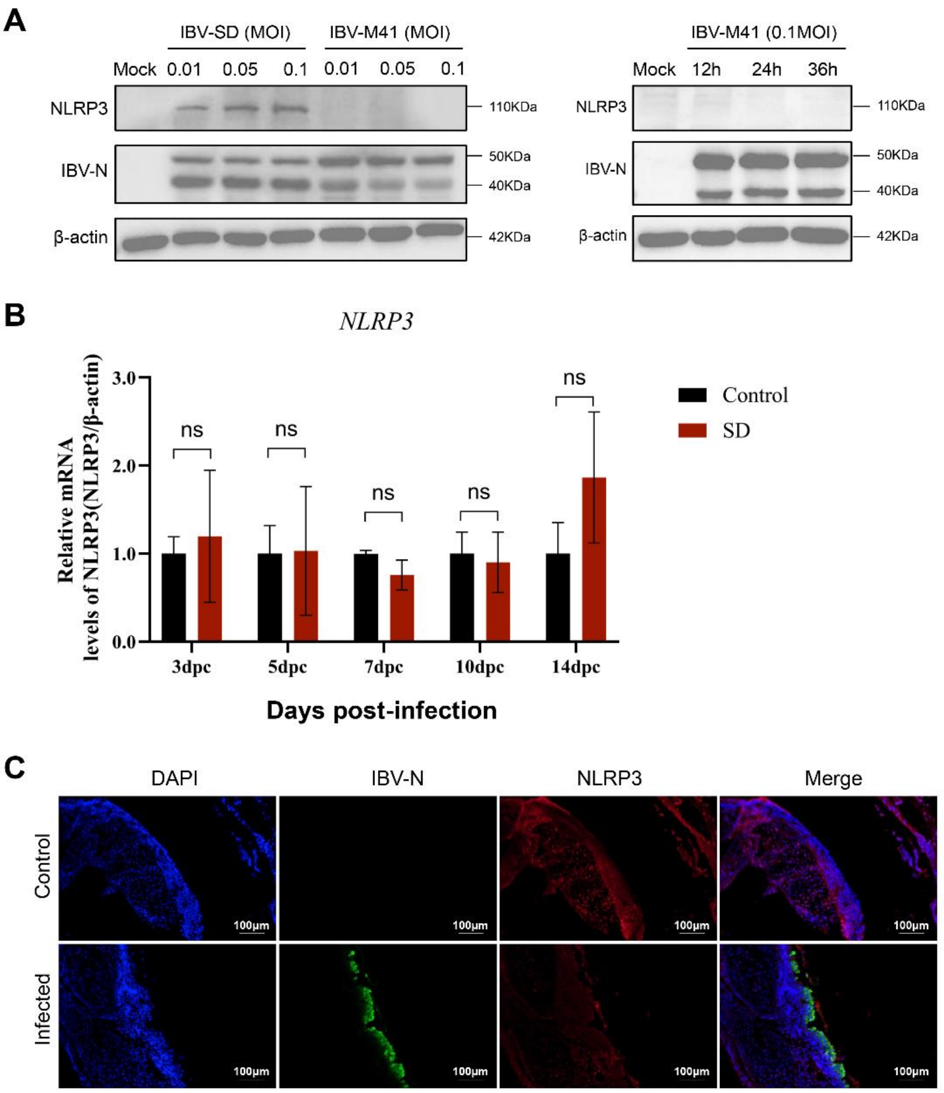

FIG S2 NLRP3 expression in IBV-infected cells and tracheal tissues. (A) Western blot analysis of NLRP3 protein levels in CEK cells infected with the IBV M41 strain at MOIs of 0.01, 0.05, and 0.1 for 24 h, and at 12, 24, and 36 h post infection (MOI = 0.1). β-actin served as the loading control. (B) qPCR analysis of NLRP3 mRNA levels in tracheal tissues collected from IBV-infected chickens at 3, 5, 7, 10, and 14 days post infection. (C) Dual immunofluorescence staining of paraffin-embedded tracheal sections at 5 days post infection, followed by confocal microscopy to visualize co-localization of IBV-N (green) and NLRP3 (red) in tracheal epithelial cells. Nuclei were counterstained with DAPI (blue).

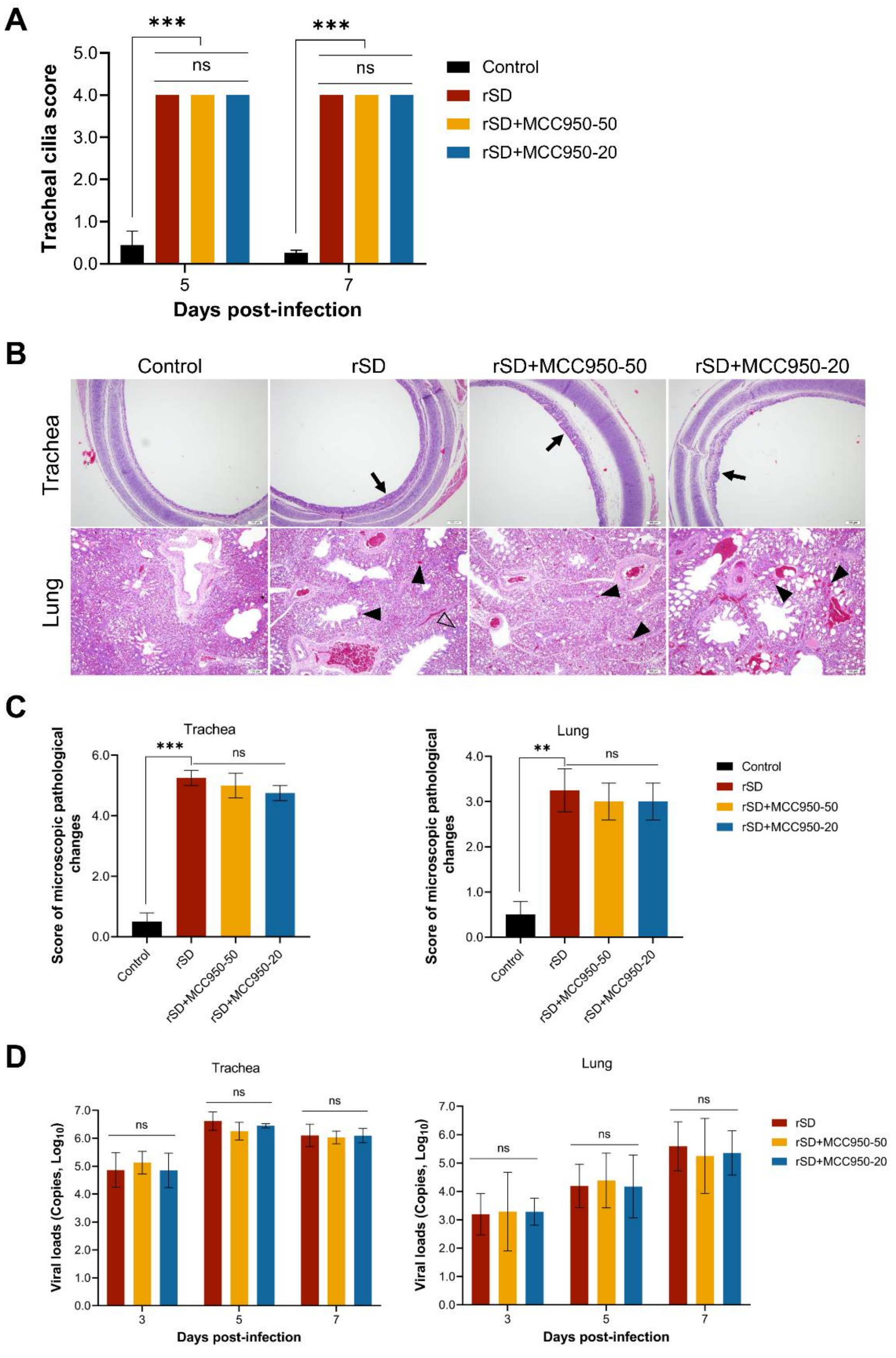

FIG S3 Tracheal and lung lesions and viral loads in IBV-infected chickens treated with MCC950. (A) Tracheal ciliary activity in chickens infected with IBV and treated with MCC950 or vehicle control. Ciliary activity was assessed using a standardized scoring system: 0 (full ciliary movement throughout the tracheal section), 1 (75–100% of

normal movement), 2 (50–75%), 3 (25–50%), and 4 (<25%). Average ciliostasis scores were calculated for each group to quantify the extent of tracheal epithelial impairment. (B) Histopathological changes in the trachea and lung, as shown by H&E staining. Black arrows indicate tracheal mucosal thickening, epithelial exfoliation and inflammatory cell infiltration. Black triangles denote mild pulmonary hemorrhage, while hollow triangles indicate limited inflammatory infiltration. No significant difference was observed between the IBV-infected and MCC950-treated groups. (C) Microscopic pathological scoring. The microscopic pathological changes were scored as follows: 0 (no microscopic lesions), 1–3 (mild lesions), 4–6 (moderate lesions), and 7–10 (severe and extensive lesions). (D) Viral loads in the trachea and lung determined by qRT-PCR targeting viral RNA.

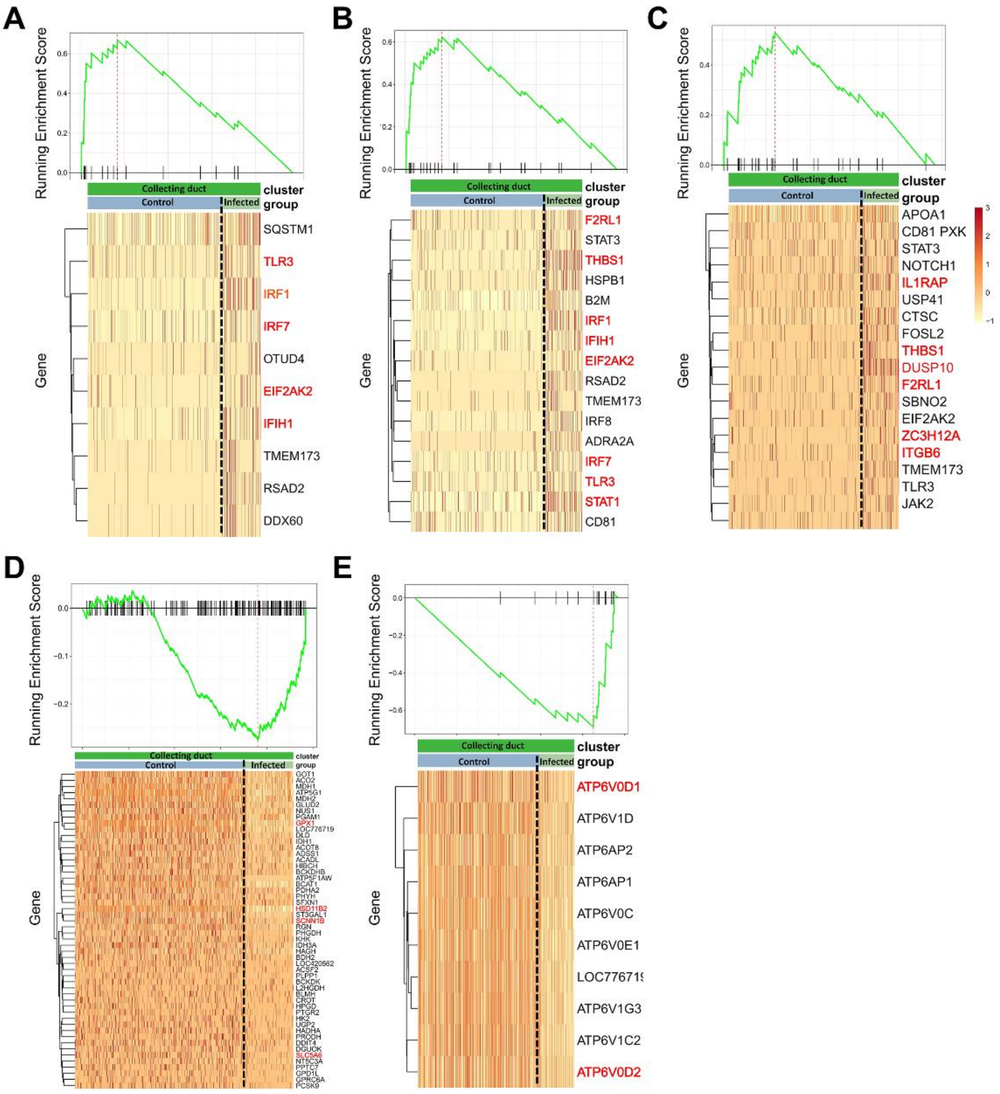

FIG S4 scRNA-seq analysis of collecting duct cells from chicken kidneys at 5 days post-IBV infection, including and heatmaps of key genes. The upper panels show GSEA results identifying significantly altered signaling pathways between infected and control groups, including: pattern recognition receptor signaling pathway (A), positive regulation of cytokine production (B), inflammatory response (C), small molecule metabolic process (D), and proton-transporting V-type ATPase complex (E). The lower panels display heatmaps of representative genes within these pathways, comparing gene expression levels in control cells (left of the dashed line) and infected cells (right). Color intensity reflects the expression level of each gene in individual cells.
